# Eszopiclone *versus* zopiclone in the treatment of insomnia

**DOI:** 10.6061/clinics/2016(01)02

**Published:** 2016-01

**Authors:** Luciano Ribeiro Pinto, Lia Rita Azeredo Bittencourt, Erika Cristine Treptow, Luciano Rotella Braga, Sergio Tufik

**Affiliations:** Universidade Federal de São Paulo (UNIFESP), Departamento de Psicobiologia, São Paulo/, SP, Brazil

**Keywords:** Insomnia, Zopiclone, Eszopiclone, Polysomnography

## Abstract

**OBJECTIVE:**

To determine the therapeutic effects of two selective GABA-A agonists, zopiclone and eszopiclone, in the treatment of insomnia.

**METHODS:**

This study comprised a phase III, single-center, randomized, double-blind, double-dummy, parallel-group, non-inferiority trial. Patients were randomized to receive zopiclone 7.5 mg or eszopiclone 3 mg, both orally, for four weeks. In total, 199 patients were evaluated during two visits and then followed for at least six weeks. The primary endpoint was the Insomnia Severity Index after four weeks of treatment. Secondary endpoints were obtained through polysomnography data, including total sleep time, sleep latency and sleep efficiency. The frequency of adverse events was also analyzed. ClinicalTrials.gov: NCT01100164.

**RESULTS:**

The primary efficacy analysis demonstrated the non-inferiority of eszopiclone over zopiclone. Analysis of objective parameters assessed by polysomnography showed that eszopiclone increased total sleep time and also improved sleep efficiency. The safety profile of both study treatments was similar and the most common events reported in both groups were dysgeusia, headache, dizziness, irritability and nausea. Adverse events were observed in 223 patients, 109 (85.2%) in the eszopiclone group and 114 (87.7%) in the zopiclone group.

**CONCLUSION:**

Based on the Insomnia Severity Index at the end of four weeks of treatment, eszopiclone demonstrated efficacy comparable to that of zopiclone in the treatment of insomnia, increasing total sleep time as well as sleep efficiency according to polysomnography.

## INTRODUCTION

Chronic insomnia affects 15% of the population 1. Although the health consequences can be severe, few patients with this disorder are diagnosed and treated appropriately. In addition to negative impacts on a wide range of daytime functions, affecting social, emotional and physical domains, chronic insomnia affects cognitive and physical functioning 2. Indeed, compared with people who do not suffer from insomnia, those who present this affliction are more prone to accidents and have higher rates of work absenteeism, decreased work performance, decreased quality of life and increased use of health care resources 3,4. Successful treatment of insomnia depends on a correct diagnosis, appropriate behavioral measures, and particularly, the use of safe and effective drugs.

Several risk factors associated with higher chronic insomnia prevalence include advanced age, female sex and the presence of comorbidities and psychiatric disorders. In fact, approximately 40% of adults with insomnia also have a diagnosable psychiatric disorder, especially depression and anxiety 3,.

The diagnosis is essentially clinical and based primarily on a detailed medical history, with some additional tools for corroboration, such as sleep diaries, actigraphy and polysomnography. The impact of insomnia on the quality of life of affected individuals has been widely studied. Drugs used to treat insomnia include hypnotics or sleep inducers as well as antidepressants with a sedative effect 8. Among hypnotics, sleep inducers with selective action on GABA-A receptors, such as zolpidem, zopiclone, eszopiclone and zaleplon are common (9,10).

Eszopiclone, a stereoisomer of zopiclone, is a non-benzodiazepine hypnotic agent of the cyclopyrrolone family. Similar to zopiclone, eszopiclone is a synthetic compound shown to be effective in treating insomnia 11-13. The selectivity of cyclopyrrolones provides greater benefits compared to benzodiazepines, as the former sustains the hypnotic effect without producing significant anxiolytic and/or muscle relaxation effects (10). The efficacy of eszopiclone has been proven in patients with insomnia associated with other comorbidities, such as a high degree of depression, generalized anxiety, rheumatoid arthritis, and sleep apnea, for which changes in sleep parameters are often observed 11,12. To date, there are no studies directly comparing the efficacy of eszopiclone and zopiclone. However, in a study of a method for assessing dissipation of the residual hypnotic effects of both drugs, a post hoc parametric analysis of reciprocal-transformed data favored eszopiclone over racemic zopiclone 14. Approved by the Food and Drug Administration (FDA, the North-American regulatory agency) in 2004, indications for eszopiclone in the treatment of insomnia are not limited to its short-term use, as its efficacy and safety have also been demonstrated in dosing studies of six to twelve month duration.

This study, a Phase III, double-blind, single-center, non-inferiority trial sponsored by Eurofarma Laboratórios S.A., aimed to determine the non-inferiority of eszopiclone (3 mg, Eurofarma) with respect to zopiclone (7,5 mg, Imovane®, Sanofi-Aventis) in the treatment of chronic insomnia.

## METHODS

Patients with symptomatic insomnia for at least three months were recruited in different ways, largely via media and a patient database. Patients between 20 and 64 years old with complaints of insomnia were selected at a screening visit (SV). Diagnosis of insomnia was established according to the criteria of the Diagnostic and Statistical Manual of Mental Disorders, fourth edition (DSM-IV) 15. An exception was with regard to the onset of symptoms because an onset of over three months was considered; thus, patients with chronic insomnia were included. Furthermore, initial polysomnography (PSG) performed no more than 90 days before the SV showing a total time of sleep of less than 6:30 h was also considered a selection criterion. In addition to the SV, the study included a baseline visit (BV; 14±3 days after the SV), an evaluation visit (V1; 14±3 days after BV) and a final visit (FV; 14±3 days after V1).

Exclusion criteria were as follows: the presence of other sleep disorders, such as sleep-wake rhythm disorders and obstructive sleep apnea with a respiratory disorder index greater than 10/hour and the presence of periodic movements of lower limbs over 15/hour; patients who were taking psychotropics and antihistamines for at least three days prior to study enrollment, hypnotics for less than 15 days, or herbal medicines or melatonin for less than 14 days; the use of hepatically metabolized drugs; a history of drug or alcohol (ethanol) use equivalent to 35 g of alcohol/day; the presence of severe comorbidities or psychiatric conditions; and patients who were pregnant or lactating or who planned to become pregnant.

A second polysomnography was performed at the end of the study, just before FV using an EMBLA polygraph. The analysis included electroencephalogram, electrooculogram, electromyogram of muscles in the chin region and the anterior tibialis, respiratory sensors (pressure cannula and thermistor), thoracic and abdominal belts, snoring and position sensors and oximetry. Events were classified according to the guidelines of the American Academy of Sleep Medicine (AASM) 16.

The main assessment tool was the Insomnia Severity Index (ISI)^17^, a questionnaire consisting of five questions and some sub-items, with replies graded from zero (best case) to four (worst case). ISI is calculated by adding the scores for each question, ranging from 0 to 28.

ISI was completed at BV, followed by randomization. All volunteers were randomized in a 1:1 ratio to receive zopiclone 7.5 mg or eszopiclone 3 mg, both orally, at bedtime.

The patients were evaluated at another two visits to the research site (visit 1 and FV) for medical history, physical examination and sleep diary evaluation; the use of concomitant drugs and frequency of adverse events were also assessed during these visits. A second polysomnography was performed immediately before FV, after visit 1. The follow-up period for each patient lasted at least six weeks.

Primary analysis of efficacy was achieved by evaluating the non-inferiority of eszopiclone in relation to zopiclone according to the ISI at the end of treatment. Secondary variables were sleep-related parameters obtained from nocturnal polysomnography and sleep-related data collected during clinical visits through the Pittsburgh Sleep Quality Index (PSQI) questionnaire 18.

The study protocol was approved by the Ethics Committee of UNIFESP/EPM and was conducted according to local regulations and good clinical practice. All patients participating in the study signed an informed consent form (ICF). This study was registered at ClinicalTrials.gov with the number NCT01100164.

### Statistical methods

The statistical software R (version 2.13.1) and MedCalc (version 11.3.3.0) were used for the statistical analysis. Continuous variables were summarized via variation (minimum and maximum values) as well as the mean, standard deviation (SD), median and interquartile range (IIQ: 25th percentile and 75th percentile). Categorical variables are described by relative frequencies.

Parametric or non-parametric methods were used for comparisons between groups according to the distribution pattern of quantitative variables in the sample. The Kolmogorov-Smirnov test with Lilliefors correction was used to assess the pattern of distribution of the endpoint variables in the sample. Lilliefors correction was also used to adjust the estimated population parameters (the mean and variance or standard deviation).

Continuous variables with a normal distribution were compared using t-tests, whereas variables with a non-normal distribution were compared using the non-parametric Mann-Whitney test. Categorical variables were compared using the chi-square test of equal proportions. ANOVA test with repeated measures was also used for comparisons between groups over time. As a general rule, two-sided 5% levels of significance were used as indicators of significant differences between the groups.

FV values were used to calculate the ISI at the end of treatment. The Kolmogorov-Smirnov test (with Lilliefors correction) showed that the variable of interest was not normally distributed in the analyzed sample. Thus, the non-parametric Mann-Whitney test was used to compare the median ISI at FV in the two treatment groups. The mean total sleep time (TST) for the polysomnography performed between visit 1 and FV was also compared in the per-protocol (PP) population by the t-test.

Sample size calculation in the proposed non-inferiority design was carried out by assuming that the ISI score after 4 weeks of treatment would be at least equal in both groups. This was based on the literature, considering an average ISI score of 9.2 points with a standard deviation of 5.7 points after four weeks of treatment for the control group. By considering an alpha-tailed error of 5% and a statistical power of 80% for the study to find the maximum difference of less than 20% between the groups (non-inferiority limit), it was estimated that 120 patients should be included in each study arm. Assuming follow-up loss of approximately 10% of patients, the study should include 130 patients in each arm, for a total of 260 patients.

## RESULTS

In total, 262 volunteers assessed at the BV and 199 were found to be eligible for enrollment: 102 in the zopiclone group and 97 in the eszopiclone group. The demographic characteristics of the patients are shown in Table 1. There were no significant differences between groups.

Of the 262 randomized patients (131 in each group), 1 patient from the eszopiclone group was excluded due to eligibility concerns, and 3 patients from the eszopiclone group and 4 from the zopiclone group were excluded due to a lack of endpoint data. Additionally, one from each group was excluded because the patient did not receive the respective drug. The ITT (intention to treat) population was composed of 126 patients in each group and the PP population included 102 and 97 patients in the zopiclone and eszopiclone groups, respectively. There were 56 premature discontinuations during the study; the causes are described in Table 2.

### Efficacy assessment

#### Insomnia Severity Index

The primary analysis of efficacy was performed by evaluating the non-inferiority of eszopiclone with respect to zopiclone based on the ISI at the end of treatment. The mean ISI reported at SV, BV, visit 1 and FV were 18.07±3.84, 16.08±4.35, 6.25±5.14 and 7.41±4.95, respectively, for patients treated with eszopiclone.

The mean ISI reported at SV, BV, visit 1 and FV were 17.86±4.08, 15.22±5.03, 7.07±4.91, 7.84±5.35, respectively, for patients treated with zopiclone. When the groups were compared regarding ISI values at FV, no statistically significant difference was observed between the treatments (*p*=0.588).

The primary analysis of efficacy in the PP population (eszopiclone *versus* zopiclone, n=199) demonstrated an upper limit of the 90% confidence interval (0.786) below the non-inferiority margin (M=1.567) developed during the sample size calculations.

### Pittsburgh Sleep Quality Index (PSQI)

Overall PSQI scores at FV were compared between the two treatment groups in the PP population. No difference between the groups regarding overall PSQI score was observed (*p*=0.2486).

### Polysomnography

At the end of the study, a significant difference between the zopiclone and eszopiclone groups regarding total sleep time was found (*p*=0.039), with a longer duration observed in the latter (Table 3). A difference between the groups (*p*=0.018) was also observed for sleep efficiency (SE), indicating greater values in the eszopiclone group (mean sleep efficiency of 90% for eszopiclone *versus* 86% for zopiclone) (Table 3). However, there was no difference between the two groups regarding sleep latency (SL, *p*=0.151) and time awake (TA, *p*=0.097) (Table 3).

### Adverse events

Adverse events were observed in 223 patients, 109 (85.2%) in the eszopiclone group and 114 (87.7%) in the zopiclone (*p*=0.552). The most frequent adverse events were dysgeusia (eszopiclone: 65 [50.78%]; zopiclone: 78 [60%]), headache (eszopiclone: 39 [30.47%]; zopiclone: 45 [34.62%]), dizziness (eszopiclone: 21 [16.41%]; zopiclone: 12 [9.23%]), irritability (eszopiclone: 9 [7.03%]; zopiclone: 12 [9.23%]), and nausea (eszopiclone: 9 [7.03%]; zopiclone: 8 [6.15%]). Nonetheless, there was no statistically significant difference between the groups in the frequency of these adverse events.

## DISCUSSION

Zopiclone is a cyclopyrrolone that differs from zolpidem by acting on the α1 and α2 subunits of GABA-A receptors and presenting a half-life of 5.3 hours. The drug has demonstrated efficacy in the treatment of early chronic insomnia or sleep maintenance and is well tolerated by the elderly; the recommended dose is 3.75 mg to 7.5 mg 8. Eszopiclone may be used for sleep maintenance insomnia 19. This cyclopyrrolone is also the first selective agonist tested in a long-term setting (six to twelve months), with reported benefits of improving quality of life, reducing workplace absenteeism and decreasing the severity of insomnia 11-13. In such cases, the recommended dose is 1 mg to 3 mg at bedtime.

This study is the first phase 3 clinical trial comparing eszopiclone to zopiclone. The primary analysis of efficacy performed considering the primary endpoint in the PP population demonstrated the non-inferiority of eszopiclone with respect to zopiclone. This is because the upper limit of the 90% confidence interval (CI) for the difference between ISI means at FV for both groups (0.785) was less than the previously established non-inferiority margin (20% of the ISI mean in the zopiclone group, i.e., 1.567). In an exploratory analysis, the 95% CI for the difference between the means was also calculated, confirming the non-inferiority of eszopiclone compared to zopiclone.

Secondary analysis of efficacy revealed significant differences in total sleep time (*p*=0.039) and sleep efficiency (*p*=0.018), as measured by polysomnography performed before and at the end of treatment; such results indicate the greater efficacy of eszopiclone in relation to these secondary variables. At the time of polysomnography, all patients were being treated with zopiclone or eszopiclone for at least 14 days, and the tests were conducted as close as possible to FV. However, intra- and/or intergroup differences may eventually have occurred due to a possible different timing of polysomnography. Because the present study focused on the clinical response of insomnia severity to eszopiclone and zopiclone, blood and urine tests or other markers were not analyzed.

The safety profile of both study treatments was similar. No statistically significant differences between the treatments were found for adverse events that were recorded in both groups and could be compared with regard to frequency. The most common events reported for both study treatments were dysgeusia, headache, dizziness, irritability, and nausea. The adverse effects of zopiclone are similar to zolpidem, including headache, dizziness and somnolence 19. Nonetheless, dysgeusia appears to be the most commonly found adverse event for both zopiclone and eszopiclone.

This study has some limitations. Although 96.2% of the patients were included in the ITT analysis, only 76% were included in the PP analysis. Patients with severe comorbidities or those using other sleep-inducing drugs, which is very common in cases of insomnia, were not included. In addition, the duration of insomnia was not quantified (even though every patient was diagnosed with chronic insomnia), and the period evaluated comprised only 4 weeks. Finally, because they were related in 2-week intervals, adverse events may have been underestimated.

Eszopiclone is effective in the treatment of insomnia, improving the severity of insomnia and demonstrating a favorable safety profile. Treatment with eszopiclone also resulted in longer total sleep time and greater sleep efficiency by polysomnography than zopiclone. However, further studies may be required to validate the polysomnographic results.

## AUTHORS CONTRIBUTION

Pinto Jr LR designed and performed experiments, analyzed data and wrote the paper. Bitencourt LR and Treptow EC designed and performed experiments. Braga LR developed analytical tools. Tufik S designed experiments, analyzed data and wrote the paper.

## FINANCIAL SUPPORT DISCLOSURE

This study was sponsored by Eurofarma Laboratórios S.A.

## OFF-LABEL INVESTIGATIONAL STUDY DISCLOSURE

This study is not an off-label investigational study.

## CONFLICT OF INTEREST DISCLOSURE

The authors report that this study received financial support from Eurofarma Laboratórios S.A to analyze an investigational drug.

## Figures and Tables

**Table 1:** Demographic and clinical characteristics of the patients in the study population (n=199).

	Study population		
Characteristic	Eszopiclone (n=102)	Zopiclone (n=97)	
Age, years (mean ± SD) [range]	48.2±10.6 [25-64]	46.5±12.1 [20-64]	
Female Sex, n (%)	76 (74.5)	73 (75.3)	
Race, n (%)			
White	79 (77.5)	72 (74.2)	
Black	8 (7.8)		14 (14.2)
Oriental	2 (2.0)	4 (4.1)	
Brown	13 (12.7)	7 (7.2)	
BMI, kg/m^2^ (mean ± SD) [range]	27.6±4.7 [18.2-40.2]	27.0±4.6 [16.1-39.0]	
Drug allergy history, n (%)	14 (13.7)	12 (12.4)	

SD: standard deviation; BMI: body mass index.

**Table 2:** Causes of premature discontinuation (n=56).

Cause	Eszopiclone n (%) n=25	Zopiclone n (%) n=31
Comorbidity	1 (4.0)	-
Non-adherence to the protocol or treatment	5 (20.0)	4 (12.9)
Follow-up loss	1 (4.0)	2 (6.4)
Refusal to continue treatment	3 (12.0)	4 (12.9)
Withdrawal of consent	2 (8.0)	2 (6.4)
Intolerance to study medication	9 (36.0)	15 (48.4)
Other sleep-affecting drug use	4 (16.0)	4 (12.9)

**Table 3:** Intergroup comparison of polysomnography carried out between visit 1 and the final visit in the eszopiclone and zopiclone groups.

	V1			VF		
	Eszopiclone (n=102)	Zopiclone (n=97)	*p*	Eszopiclone (n=102)	Zopiclone (n=97)	*p*
*TST (minutes)*						
Median (IIQ)	332.0 (301.5-355.75)	315.5 (276.5-356.0)	ns	377.0 (343.6-413.9)	369.0 (338.0-387.5)	0.0396
*SE (%)*						
Median (IIQ)	81.2 (69.6-86.2)	77.5 (68.2-85.4)	ns	90.1 (84.2-93.02)	86.0 (81.2-91.57)	0.01802
*SL (minutes)*						
Median (IIQ)	15.4 (6.8-31.0)	20.4 (9.2-40.3)	ns	8.8 (3.7-19.4)	14.2 (3.4-29.4)	0.1514
*TA (minutes)*						
Median (IIQ)	62.5 (39.3-87.5)	62.1 (39.8-98.9)	ns	30.2 (16.0-48.8)	39.0 (20.2-64.6)	0.0974

Visit 1 (V1) and the final visit (VF) TST: total sleep time; SE: sleep efficiency; SL: sleep latency; TA: time awake; IIQ: interquartile range; ns: non-significant.
